# Assessment taxonomy and pathways of alien plant species in Egyptian protected areas

**DOI:** 10.1038/s41598-025-24266-3

**Published:** 2025-11-12

**Authors:** Salma K. Shaltout, Elbialy E. Hatab, Mohamed M. El-Khalafy, Yassin M. Al-Sodany, Amr E. Keshta

**Affiliations:** 1 Botany Department, Faculty of Science, Tanta University, Tanta, 31527 Egypt; 2 Biodiversity Division, Nature Conservation Sector, Egyptian Environmental Affairs Agency, Cairo, Egypt; 3 Botany and Microbiology Department, Faculty of Science, Kafrelsheikh University, Kafrelsheikh, Egypt; 4 Smithsonian Environmental Research Center, Edgewater, Maryland USA

**Keywords:** Alien species, Protected areas, Invasive plant species, Habitat, Wetlands, Egypt, Ecology, Ecology, Environmental sciences

## Abstract

Invasive species are a real threat to biodiversity worldwide, and Egypt is no exception for that threat. In Egypt, there is thirty protected areas, representing most of the ecological important areas in Egypt, is declared and managed by the Nature Conservation Sector Agency, Egypt, with an approximate area of 142,664 km^2^ (representing 14.3 % of Egypt’s country area). Until 2012, the declared protectorates include the following four groups: 7 marines, 7 wetlands, 10 deserts and 6 geological protectorates. The goals of the present study were: 1) creating an inventory of alien species in the Egyptian protected areas, 2) assessing the protection effect on the spread of invasive species in different protected areas, and 3) assessing the pathway of alien species into the protected areas and how to control them. This study was conducted by reviewing current and available literature, field trips, and herbaria consultation. Our results indicated that Lake Burullus has the highest number of alien species (27= 13.7%), while Abu-Galum has the lowest (2= 1.2%). On the other hand, some protected areas (e.g. Nabq, Wadi Degla, White Desert, El-Gilf El-Kebir) have no alien species. This indicates that some protected areas are experiencing high levels of protection, and their locations are less affected by human activities. Three non-native invasive species (*Azolla filiculoides, Bassia indica*, and* Eichhornia crassipes*) are threating the ecosystem of Ashtum El-Gamil, Wadi El-Rayan and Lake Burullus Qaroun. On the other hand, however, *Ziziphus spina-christi* was recorded in seven protected areas as sand controllers and has medicinal and grazing benefits. Among the three different categories of the alien species, the naturalized species group has the highes According to the national strategy for biodiversity in Egypt – For a sustainable Future 2030 (CBD 2020) this study provides a comprehensive assessment of alien species dynamics in the ecosystem of Egyptian protected area.

**Supplementary Information:**

The online version contains supplementary material available at 10.1038/s41598-025-24266-3.

## Introduction

Invasive species (both plants and animals, however our focus in the current study is the invasive plant species) pose a significant threat to biodiversity and ecosystems worldwide, and Egypt is no exception. These species, often introduced through human activities including importing for ornamental purposes, can outcompete native flora, disrupt ecosystems^1–4^, and cause economic damage^5^. In Egypt, the study of Shaltout^6^, a list of the alien species includes 250 species. Pteridophyta are represented by only one family (Azollaceae) and one taxa *Azolla filiculoides*, while dicotyledonous taxa are represented by 34 families, 110 genera and 166 species. Monocotyledonous taxa are 7 families, 47 genera and 83 species. Three categories of alien species are recognized: causals (114 species), naturalizers (129 species), invaders (7 species).

Protected areas, defined by the IUCN as a clearly defined geographical space, recognized, dedicated and managed, through legal or other effective means, to achieve the long-term conservation of nature with associated ecosystem services and cultural values”^7^, are regarded as one of the most important approaches for conserving biodiversity globally^8,9^. In Africa, PAs Currently, 19% of Africa’s land and 17% of the seas around Africa are covered by protected and conserved areas^10^. The percentage of PAs relative to the entire terrestrial region of Northern Africa was about 4.0 % in 2010 (3.7 % in 2000; 3.3 % in 1990) and approximately 11.8 % in sub-Saharan Africa that same year (11.3 % in 2000; 11.1 % in 1990). However, many PAs are likely to be contested as being ‘paper parks’ (i.e. areas that have been proclaimed but have little or no management and are therefore ineffective in fulfilling their mandate^11^;).

Egypt’s protected areas, which include national parks and nature reserves, are crucial for conserving the country’s unique biodiversity. These areas, such as Sinia (especially South Sinai, SSI, which is a Saint Kathrine protected area located) have the maximum number of endemics (45.2%)^12^. However, the presence of invasive plant species within these protected areas poses a severe challenge to conservation efforts. Invasive plants can alter habitat structures, reduce the availability of resources for native species, and ultimately lead to a decline in biodiversity as has been reported in Egypt^13–15^ and around the world^1,2,4,16^.

Efforts to manage and mitigate the impact of invasive plant species in Egypt’s protected areas involve a combination of different strategies. These include mechanical removal, chemical treatments, and biological control methods. For instance, in areas heavily infested with *Azolla filiculoides*, mechanical removal is often employed to prevent the fern from clogging waterways and affecting aquatic life^13^. Additionally, public awareness campaigns and community involvement are essential components of invasive plant species management, as they help prevent the introduction and spread of these plants^17^.

Despite these ongoing efforts by the Egyptian local environmental agencies, the battle against invasive plant species in Egypt’s protected areas is ongoing. Continuous monitoring and research are necessary to understand the dynamics of invasive species and develop effective management strategies. Collaboration between governmental agencies, conservation organizations, and local communities is vital to protect Egypt’s natural heritage from the threats posed by invasive plants^17^). By addressing these challenges, Egypt can ensure the long-term preservation of its unique ecosystems and the biodiversity they support^15^.

Natural protected areas have a fundamental role in ecotourism^18,19^, safeguard for both nature and cultural resources^20^, places that offer sustainable development for long-term experimental research^21,22^, and biodiversity conservation^23^. Continuous and effective management for natural protected areas are crucial for biodiversity conservation and maintaining ecosystem services and functions. Moreover, by safeguarding these valuable protected areas, Egypt can ensure continued benefits from the ecosystem services they provide and preserve its unique natural heritage for future generations. Invasive alien species are one of the most significant threats to biodiversity worldwide, including natural reserves. These species are known for their rapid spread and dense reproduction, disrupting the ecological balance and threatening native species and fragile ecosystems.

Effective management strategies require a comprehensive understanding of the characteristics and behaviors of invasive species to implement targeted and efficient countermeasures. Additionally, accurate and prompt species identification is essential for effective invasive species management before the species becomes established Recognizing the importance of addressing invasive species in natural reserves, this study focused on: identifying all alien species present within the targeted natural reserve, the distribution of these species within the reserves was analyzed to pinpoint areas with the highest concentrations and evaluation the status of alien species within these reserves, including their density, and environmental impact.

### Study area and habitat

**Fig. 1:**
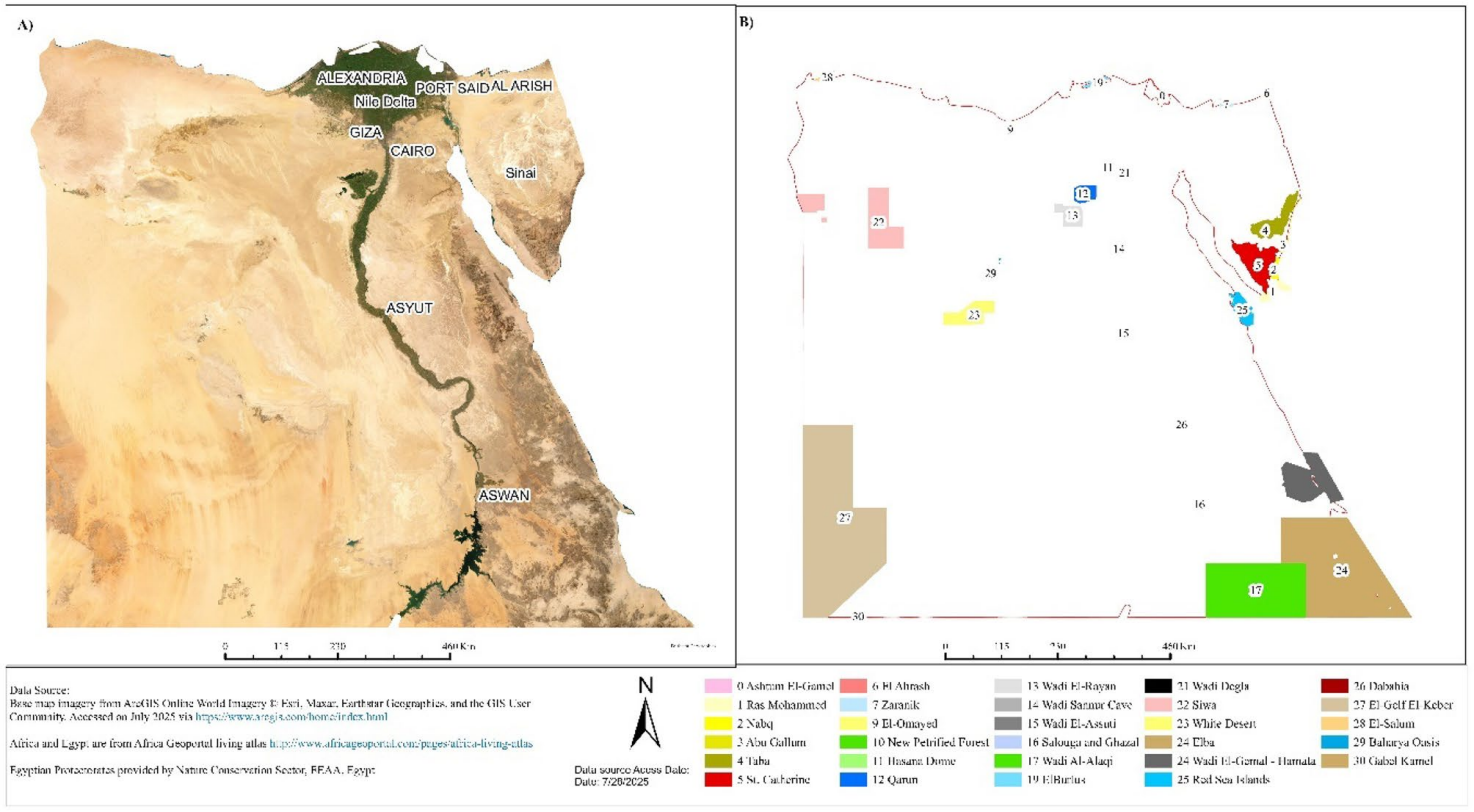
Study-site map showing satellite base imagery for Egypt **(A)**, while panel **(B)** is showing the location of the Egyptian protectorates. GIS data were accessed in July 2025, and the map was produced in ArcGIS Pro (version 3.5.2).

In Egypt, Since the passage of Law 102/1983, 30 Protected Areas have been declared. They cover almost 14.3% of the country land and marine areas and include a representative range of habitats and physiographic regions, along with other sites of importance such as biodiversity hotspots, cultural heritage sites, geological formations and landscapes of outstanding natural beauty (Fig. 1)^24^. These natural protected areas spanning different environments including marine, desert, natural geological formations, and wetland ecosystems. There are 7 marines, 10 deserts, 7 wetlands, and 6 geological protectorates. Most of the wetland and marine protectorates are located along the coast of Red and Mediterranean Sea, while desert protectorates are distributed in Sinai and West desert of Egypt. The current study assessed the current status of the alien species and their invasion status among the 18 natural protected areas that the information about flora detected (Table 1).

**Table 1:** The Protectorates under study which declared in the framework of Law 102 of year 1983^26^.

	**Protectorates**	**Declaration**	**Area** **(km**^**2**^**)**	**Governorate**	**Coordinates**	**No. of habitats**
1	Ras Muhammad	1983	850	South Sinai	27.7222°N, 34.2539°E	10
2	Abu Galum	1992	500	South Sinai	28°52′25″N, 34°27′31″E	6
3	Saint Kathrine	1988	5750	South Sinai	28°33′20″N, 33°58′34″E	6
4	Zaraniq	1985	230	North Sinai	31°06′00″N, 33°26′31″E	5
5	Ashtum El Gamil	1988	180	Port Said	31°15′35″N, 32°09′34″E	5
6	Omayed	1986	700	Matrouh	30°49′05″N, 29°09′44″E	7
7	Petrified Forest	1989	7	Cairo	29°58′49″N, 31°27′27″E	1
8	Lake Qarun	1989	250	Fayoum	29°27′13″N, 30°34′51″E	8
9	Wadi Elrayan	1989	1225	Fayoum	29°08′52″N, 30°23′33″E	7
10	Wadi Allaqi	1989	30000	Aswan	20°20′N, 32°40′E	6
11	Gabal Elba	1986	35600	Red Sea G	22°11′16″N, 36°22′14″E	13
12	Lake Burullus	1998	460	Kafr El Sheikh	31°29′N, 30°52′E	9
13	Wadi Degla	1999	60	Cairo	29°57′34″N, 31°19′54″E	2
14	Siwa Oasis	2002	7800	Matrouh	29°11′N, 25°33′E	12
15	Sallum	2007	383	Matrouh	22°11′16″N, 36°22′14″E	1

According to Harhash *et al.*^25^ Egypt have been classified into 36 habitats, however that the Protected Areas are of varying size and habitat types, from the largest, Elba, at about 35,600 km^2^ with 13 habitats (mountain, plateau, wadi, Hamada, sand dunes, Hatya, salt marshes, mangrove, sabkha, coral reefs, seagrass bed, marine (red sea, marine island) to Petrified Forest, at 7 km^2^ with only one habitat (hill/plateau) (Table 1). Until the mid-1990’s, protected areas were declared in a somewhat subjective manner rather than according to predetermined criteria. Consequently, there is insufficient representation of some habitats in the network, particularly marine habitats^26^.

## Methods

### Study area and temporal scope

Field surveys were undertaken within 15 protected areas in Egypt chosen based on their recognized importance for plant biodiversity conservation. Data collection occurred during 10 field campaigns spanning from Spring 2021 to Summer 2024.

### Species recording and presence/absence assessment

During each field campaign, the presence or absence of plant species within each protected area was systematically recorded. The recorded species lists were explicitly compared against the comprehensive checklist of alien species in the Egyptian flora published by El-Bhary et al.^27^ to identify alien taxa.

### Plant identification and taxonomic verification

**Primary Identification:**Plant specimens collected in the field were identified using standard Egyptian botanical references^28–34^:.

**Herbarium Validation:** All identifications were critically revised and verified at the Tanta University Herbarium (acronym TANE, following^16^) using authenticated reference specimens.

**Literature Cross-Referencing:**Species not encountered during field collection or lacking documentation in the primary identification literature were cross-checked against regional floristic studies and checklists, specifically^35–44^:.

**Family Classification:** Genera and species were assigned to families according to the taxonomic system in^33^.

### Supplementary data collection (expert Consultation)

An attempt was made to gather additional information through a questionnaire distributed to 30 experts working within the Egyptian protected area sector. We sought to simulate the experiment conducted by^45^ using a similar questionnaire. (supplementary 1). However, we employed Google Forms as the survey platform due to its widespread use in our region. The response rate was low (11 respondents), likely due to limited recorded information within the sector.

### Classification of alien species

Alien species identified through the above process were classified into one of three categories based on their level of establishment: **Casual**, **Naturalized** and **Invasive.** This classification followed the **definitions and conceptual framework** outlined by^6^, which are fundamentally aligned with the standardized criteria established by^46^.

### Dispersal mechanism analysis

For species where seeds were collected, the **primary dispersal type** was assessed and categorized according to the scheme proposed by^47^.

### Introduction Pathway Assessment

The pathway of introduction for the identified alien species into the protected areas were assessed using two methods: Field observations made during the survey campaigns. And Consultation of available records and expertise from the Nature Conservation Sector (Egyptian Environmental Affairs Agency). Pathways were classified and interpreted based on the standardized categories and guidance provided under the Convention on Biological Diversity^48^.

## Results

### Assessment and distribution pattern of alien species in Egyptian protected area (EPA):

**Fig. 2:**
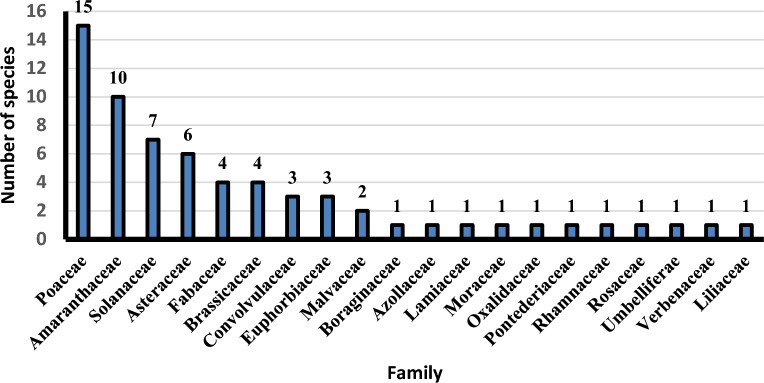
Distribution of alien taxa in families.

**Fig. 3:**
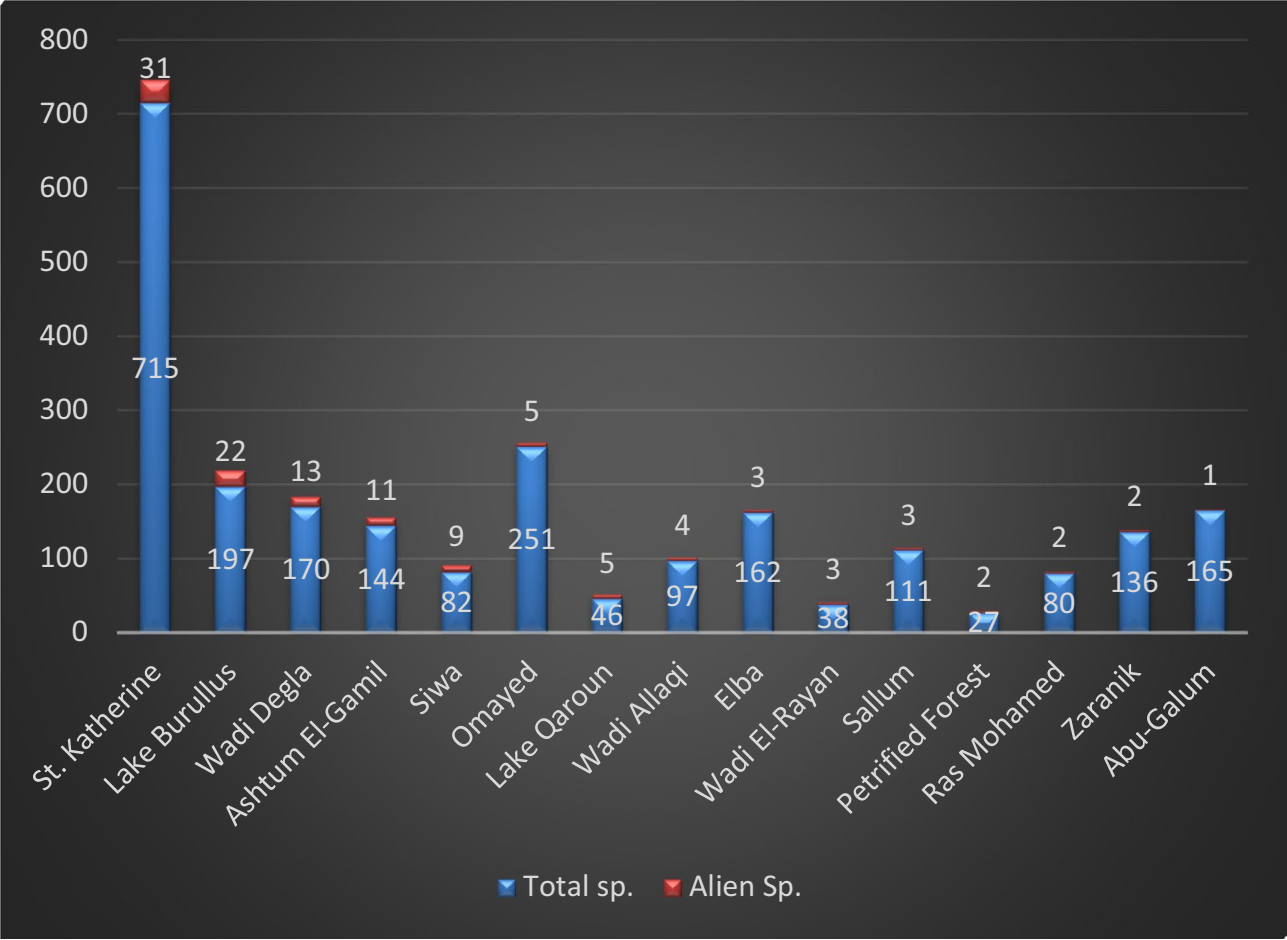
Number of total species versus alien species in 15 protected area in Egypt.

This study covers 15 protected areas from 30 total. Sixty-five alien species (26.4% of total alien sp. in Egypt) were recorded (supplementary 2), which represented by 20 families, 51 genera; Poacea (15 sp. = 23.1%) and Amaranthaceae (10 sp. = 15.4%) are the most represented followed by Solanaceae (7 sp. = 10.8%), Asteraceae (6 sp. = 9.2%), Brassicaceae and Fabaceae (4 sp. = 6.2%) and Convolvulaceae and Euphorbiaceae (3 sp. = 4.6%). On the other hand, Malvaceae are represented by 2 species, while Azollaceae, Lamiaceae, Moraceae, Oxalidaceae, Pontederiaceae, Rhamnaceae, Rosaceae, Umbelliferae and Verbenaceae by only one species (Fig 2). The recorded species are distributed as following; Sant Katrin (31 species 47.6% of total alien sp. in EPA) have the greatest number of alien species, followed by Lake Burullus (22 sp. = 33.9%), Wadi Degla (13 species = 20.0%), Ashtum El-Gamil (11 sp. = 16.9%), Siwa (9 sp. = 13.9%), Lake Qaroun (5 sp. = 7.7%) and Omayed (5 sp. = 7.7%), On the other hand, Petrified Forest and Zaranik (2 sp. = 3.1%) and Abu-Galum have only one species (*Ziziphus spina-christi*). while Nabq, White Desert and El-Gilf El-Kebir do not have any alien species (Fig. 3).

**Fig. 4:**
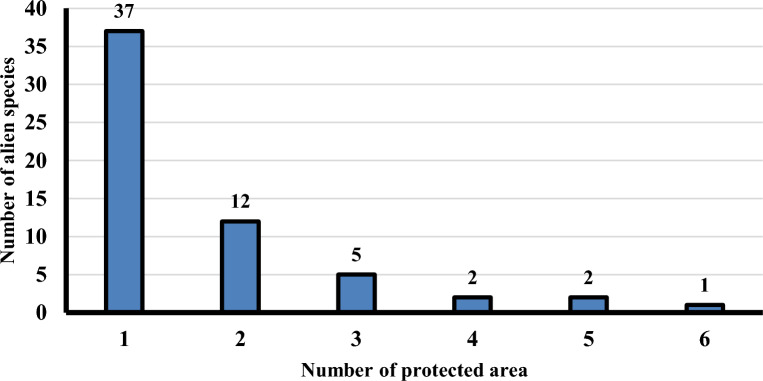
Relation between number of alien species and number of protected areas in Egypt.

Thirty seven alien species recorded on one protected area and 12 alien species recorded in two protected area, while one species recorded in six protected areas (*Bassia indica*), two species recorded in five protected areas (*Beta vulgaris* subsp. *Maritime* and *Ricinus communis*), two species recorded in four protected area (*Centaurea calcitrapa*, *Eruca vesicaria* and *Ziziphus spina-christi*), and five species recorded in three protected areas (*Arundo donax*, *Bromus catharticus*, *Dysphania ambrosioides*, *Sesbania sesban* and *Eichhornia crassipes*) (Fig. 4).

**Fig. 5:**
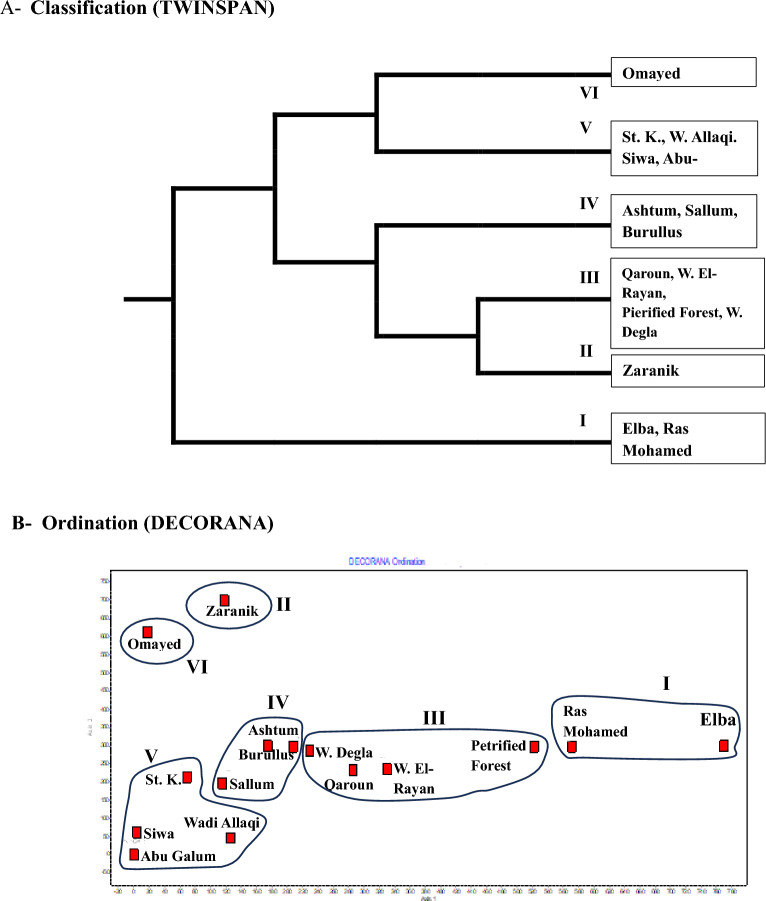
Classification (**A**) and ordination (**B**) of the 15 Egyptian protected area based on their content of alien species in the Egyptian flora. Group I: *Cenchrus ciliaris*, Group II: *Solanum elaeagnifolium*, Group III: *Bassia indica*, Group IV: *Beta vulgaris* subsp. *maritima*, Group V: *Ziziphus spina-christi* and Group VI: *Dactylis glomerata*.

Application of TWINSPAN classification to the 65 alien species in the 15 protected areas led to distinguish 6 main groups at 4^th^ level (Fig 5a). Group I: represents Ras Mohamed and Gabel Elba and dominated by *Cenchrus ciliaris and Cuscuta chinensis*, Group II: represents Zaranik and dominated by *Solanum elaeagnifolium and Centaurea calcitrapa*, Group III: represents Qaroun, Wadi El-Rayan, Petrified Forest and Wadi Degla and dominated by *Bassia indica and Ricinus communis*, Group IV represents Ashtum El-Gamil, Lake Burullus and Sallum and dominated by *Beta vulgaris* subsp. *maritima and Centaurea calcitrapa*, Group V represents Saint Katharine (St. K.), Wadi Allaqi, Siwa and Abu-Galum and dominated by *Ziziphus spina-christi and Ricinus communis*, and Group VI represents Omayed and dominated by *Dactylis glomerata* and *Lycium europaeum* (Table 2). Most of these groups are well segregated along the DECORANA ordination plane (Fig. 5b).

**Table 2:** Application of TWINSPAN classification to the 65 alien species in the 15 protected areas.

**VG.**	**First dominant**	**P (%)**	**Second dominant**	**P (%)**
**I**	*Cenchrus ciliaris* L.	100	*Cuscuta chinensis* Lam.	50
**II**	*Solanum elaeagnifolium* Cav.	100	*Centaurea calcitrapa* L.	100
**III**	***Bassia indica* (Wight) A. J. Scott**	100	*Ricinus communis* L.	50
**IV**	*Beta vulgaris* subsp. *maritima* (L.) Arcang.	100	*Centaurea calcitrapa* L.	67
**V**	*Ziziphus spina-christi* (L.) Desf.	100	*Ricinus communis* L.	75
**VI**	*Dactylis glomerata* L.	100	*Lycium europaeum* L.	100

### The status evaluation of Alien species in EPA

**Fig. 6:**
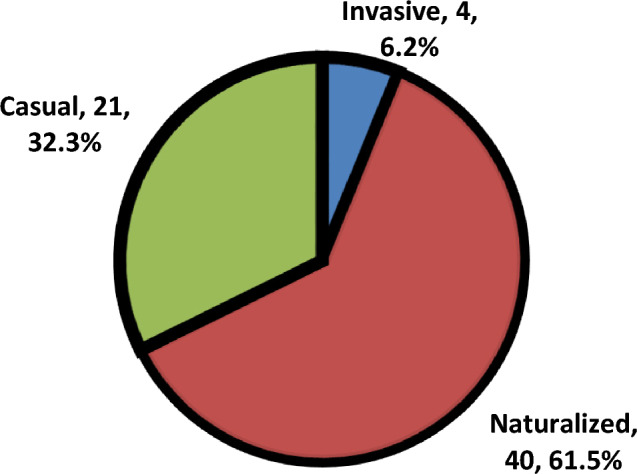
The percentage of three categories (Casual, Naturalized and invasive) of alien species.

Naturalized species (40 sp. = 31.0% of total naturalized species in Egypt)) are more represented than casual species (21 sp. = 18.4% of total casual sp. in Egypt). On the other hand, invasive species are represented by (4 sp. = 57.1% of total invasive species in Egypt). repeated species in one more protected area (5.54%) and 60 species without any repeating (Fig. 6).

### Pathway and dispersal types of Alien species in EPA

The determination of the dispersal types of alien species indicated that the microsclerochores (16.9%), Ballochore, desmochore and sarcochore (13.8% each) are the most represented. On the other hand, megasclerochore and pyrenochore (1.5%) are the lowest represented (Table 3)

**Table 3:** Dispersal types of alien species with their categories.

**Dispersal type**	**Casual**	**Naturalized**	**Invasive**	**Total**	**%**
Auxochore	1	1	3	**5**	7.7
Ballochore	2	7		**9**	13.8
Barochore	2	2		**4**	6.2
Cyclochore	2		1	**3**	4.6
Desmochore	3	6		**9**	13.8
Megasclerochore	1			**1**	1.5
Microsclerochore	4	7		**11**	16.9
Pogonochore	1	4		**5**	7.7
Pterochore	2	4		**6**	9.2
Pyrenochore	1			**1**	1.5
Sarcochore	1	8		**9**	13.8
Sporochore	1	1		**2**	3.1
**Total**	**21**	**40**	**4**	**65**	

**Fig. 7:**
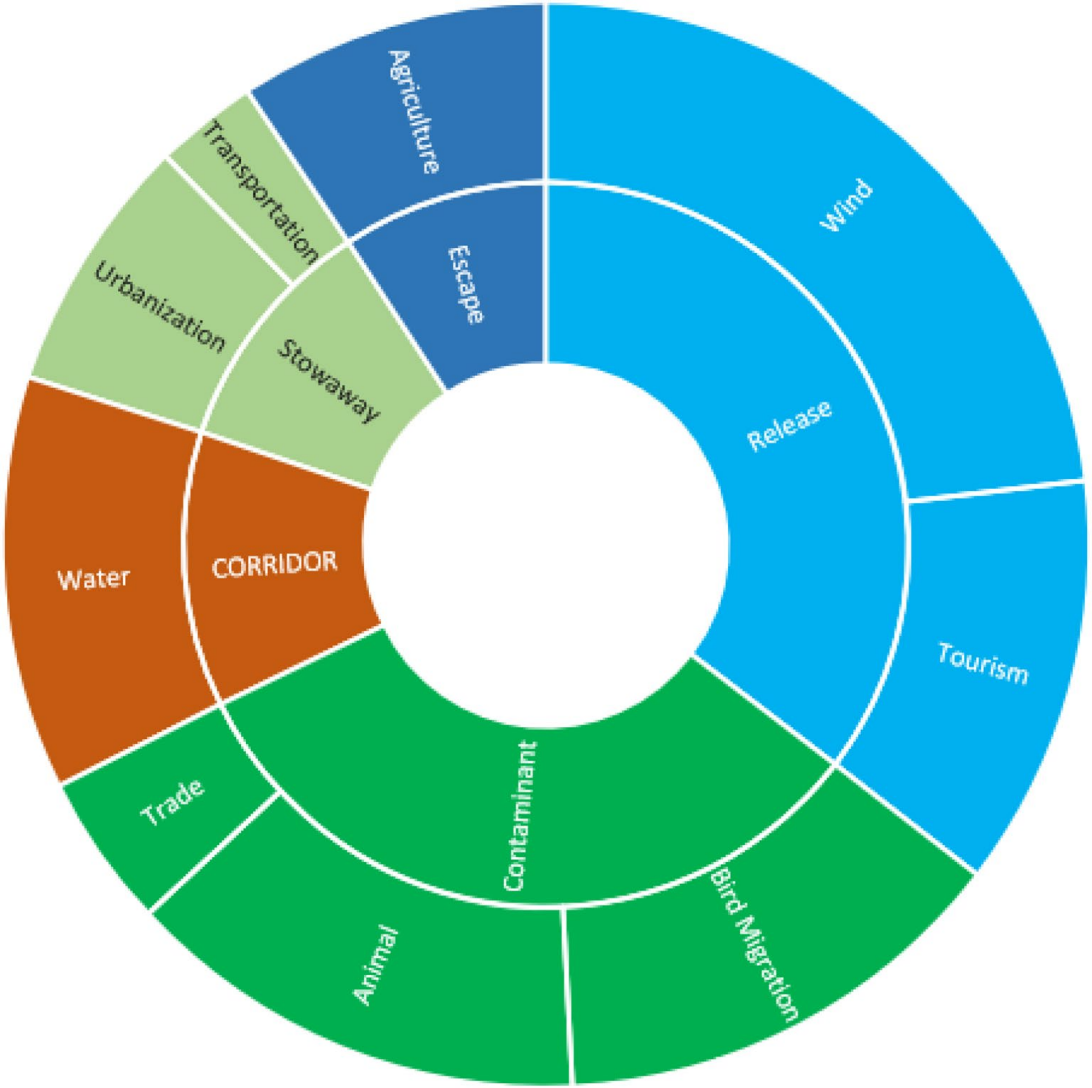
Relationship between Number of protected areas and the number of pathway categories and subcategories of alien species distribution according to CBD 2018.

In the present study, introduction pathways were classified into five main categories, further subdivided into nine subcategories. The wind was identified as the predominant pathway, reported in 15 protected areas (PAs), followed by bird migration (9 PAs) and tourism (8 PAs). In contrast, urbanization (5 PAs), trade (3 PAs), and transportation (2 PAs) were the least represented pathways (Figure 7).

These findings align with the questionnaire results, which indicated that habitat loss and fragmentation constituted the most significant threat (Rank 1). This was followed by pollution, also ranked as a primary threat (Rank 1), and overgrazing (Rank 2). Conversely, invasive species were identified as a lower-level threat, while tourism was most frequently assigned to Rank 3.

**Fig. 8:**
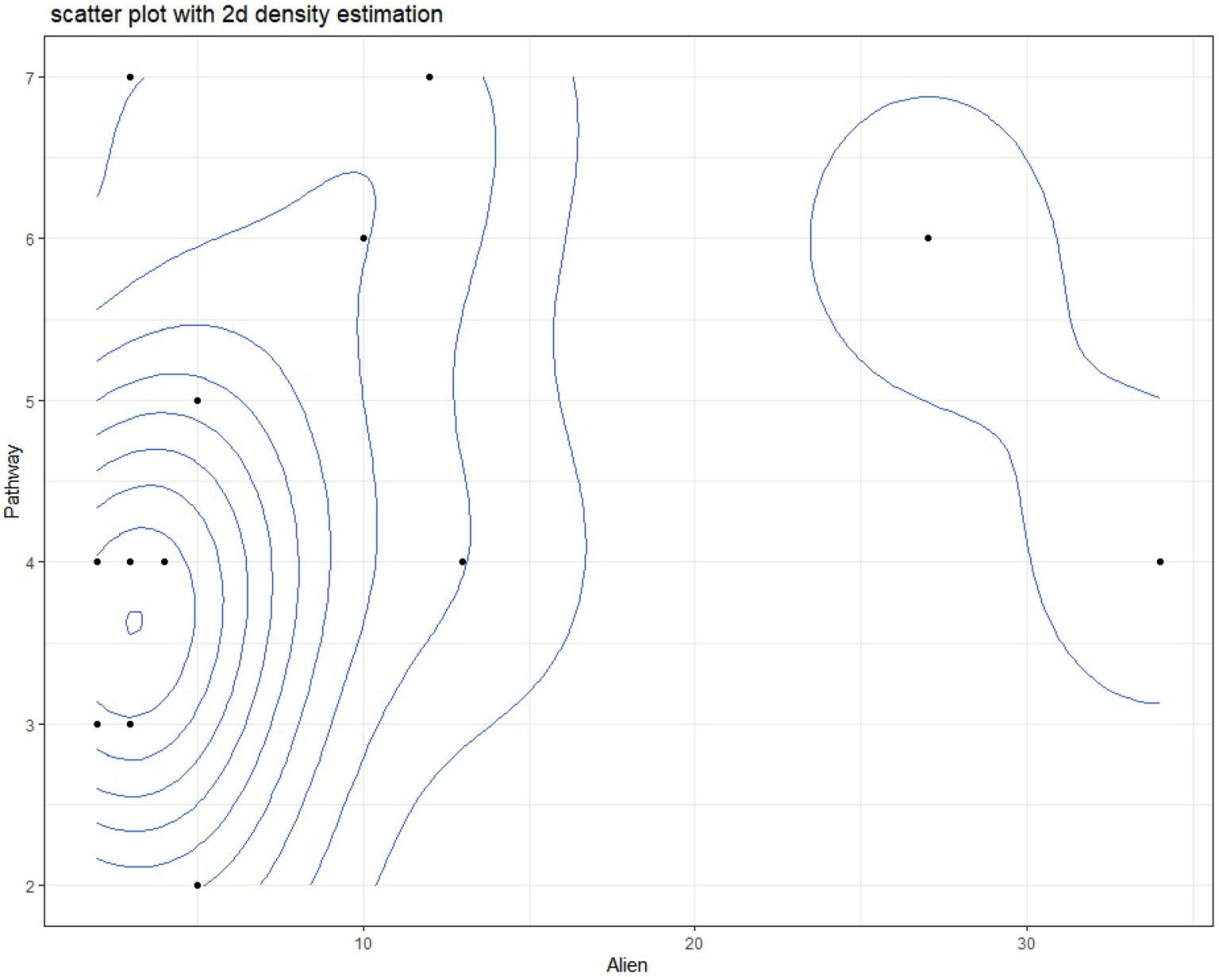
The relation between the number of subcategories pathways and the number of alien species in each EPA.

**Fig. 9:**
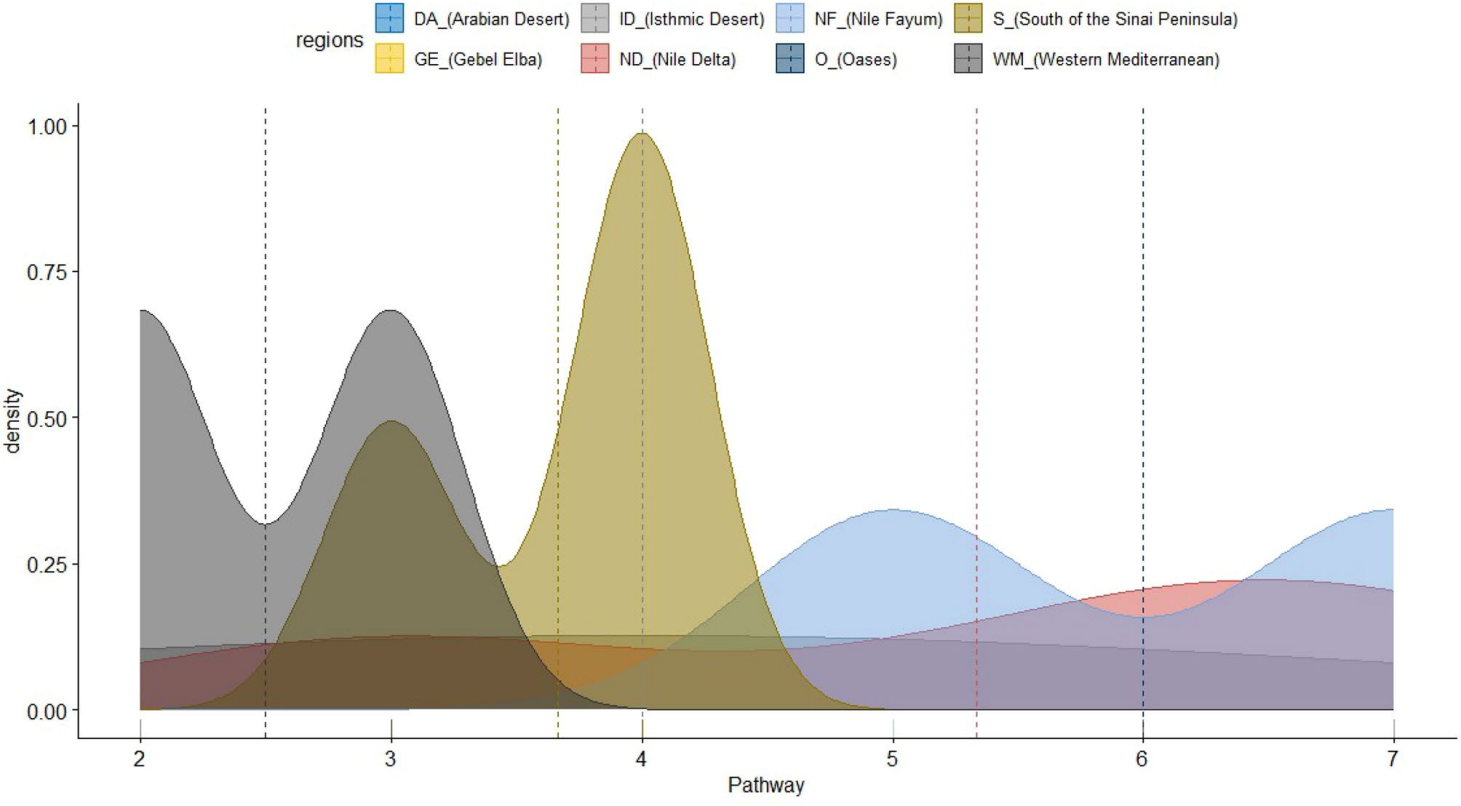
Density plot with pathway and alien plant species mean lines, categorized by the phytogeographical regions of the protected areas studied**.**

Ashtum El-Gamil and Wadi El-Rayan EPA were the most susceptible to invasion subcategories pathways, seven pathways documented in each. Followed by Lake Burullus and Siwa oasis, where 6 pathways were recorded, while El Omayed was the least affected by the invasion pathway. (Fig. 8 and 9).

**Fig. 10:**
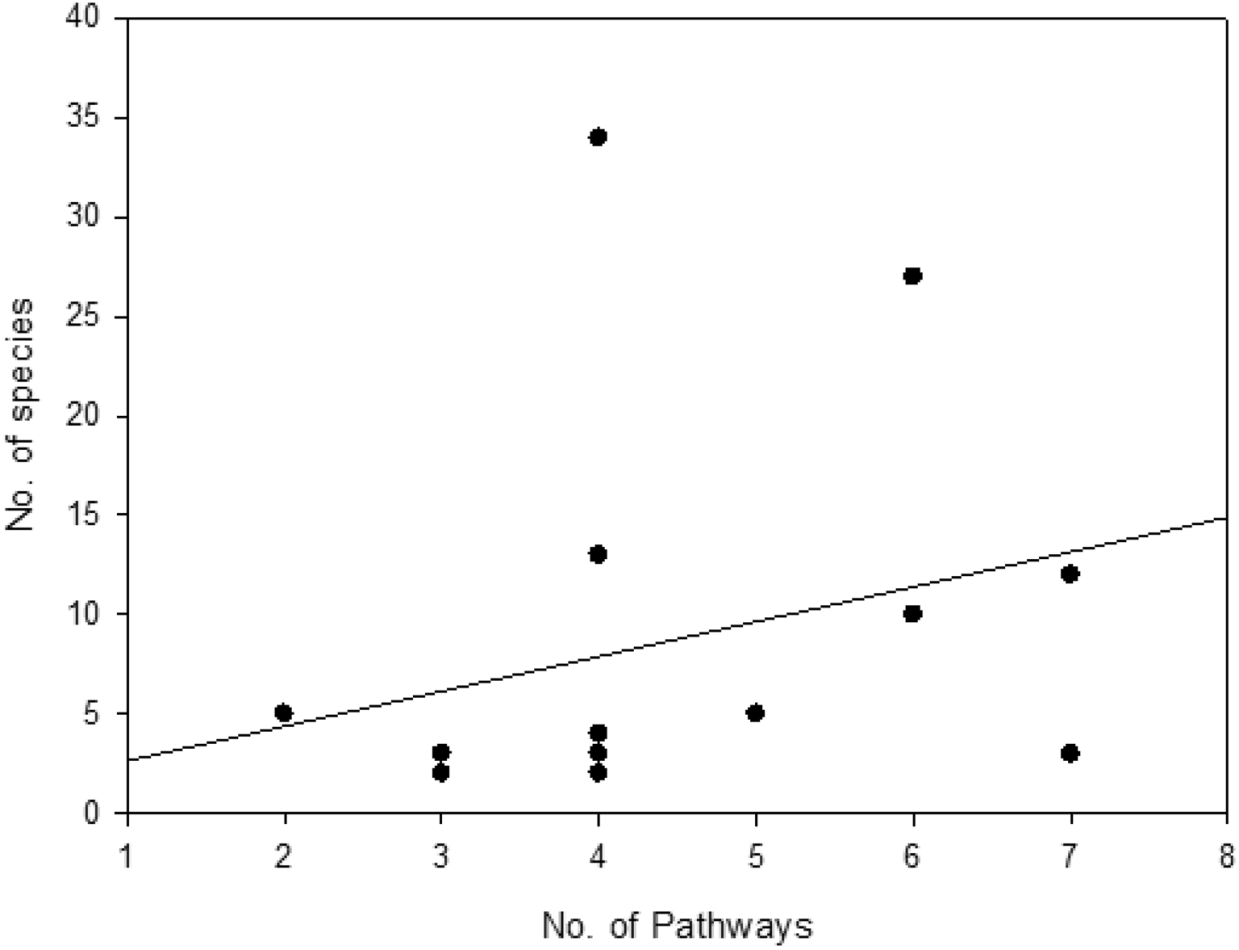
Non-significant correlation between Number of pathways and number of alien plant species.

Analysis revealed a weak positive correlation between the number of introduction pathways per protected area and the number of alien plant species recorded within it (Pearson’s r = 0.272, p = 0.326). This relationship was not statistically significant. (Fig. 10).

## Discussion

This study highlights a significant presence of alien species within Egyptian protected areas, with 65 species recorded, representing a substantial portion (26.4%) of the total alien flora in Egypt. The taxonomic composition dominated by Poaceae and Amaranthaceae, families known for their adaptability and invasive tendencies in various ecosystems, underscores the potential for these groups to establish and spread in arid environments. Pashkevych & Burda^49^ indicated that Fabaceae, Poaceae, Asteraceae and Myrtacea are very widespread invaders that have large global distributions. The high representation of alien species raises concerns about the ecological integrity of these protected areas, potentially impacting native biodiversity and ecosystem functions.

Knowledge of alien plant invasions within African PAs remains limited^50^. Our study provides a crucial initial assessment for Egypt, synthesizing field observations, published literature, and grey literature. However, this effort was constrained by incomplete baseline data; 60% of the studied PAs lacked any prior plant diversity checklist, and only 40% had partial lists including alien species prior to this work. Alien species are found in all taxonomic groups and most habitat types, in any region of the world, have been affected to some extent. In protected areas impacts from alien species consist in impact on ecosystem function and structure, furthermore at the level of species communities or habitats as well as at the level of species^49,51^.

The high species richness in areas like Saint Katherine (31 species) and Lake Burullus (22 species) may correlate with these areas being threatened by intensified human activities (e.g., tourism, agriculture) and climatic shifts that create favorable niches for alien establishment^52^. Conversely, the absence of alien species in areas such as White Desert and El-Gilf El-Kebir likely reflects their remoteness or minimal anthropogenic pressure.

TWINSPAN and DECORANA analyses grouped the protected areas into six distinct clusters, suggesting ecological or anthropogenic gradients influencing alien distribution. For instance, the clustering of Abu-Galum**,** Siwa and Wadi Allaqi with Saint Katherine may reflect shared climatic stressors^53^ or human practices (e.g., livestock grazing). These findings align with^54^ who emphasized human-mediated drivers of alien proliferation.

Naturalized species (40 species; 31.0% of Egypt’s naturalized flora) outnumbered casual (21 species; 18.4%) and invasive species (4 species; 57.1% of Egypt’s invasive flora). This dominance highlights the successful integration of many aliens into local ecosystems without reaching an invasion. However, the presence of four invasive species, notably *Bassia indica* (recorded in six areas) signals ecological risks, as their spread often correlates with habitat disturbance (e.g., agricultural or urban encroachment), consistent with^55^**.** The prevalence of naturalized species necessitates long-term monitoring programs to detect potential shifts toward invasiveness under changing environmental conditions. The status of invasive plant management of PAs has not got any assessment at all; and most of them are without any control activities. Management measures implemented on invasive alien plants showed a similar deficiency as the checklists^46^.

The initial stage of invasion involves the human-assisted movement of organisms or propagules beyond their native range via specific pathways and vectors^56^. Globalization, driving increases in transport, trade, travel, and tourism, is a key factor accelerating species movements worldwide^57–59^; enabling the crossing of biogeographical barriers. Understanding these pathways is critical for effective management.

Alien species dispersal operates across spatial scales, influenced by global transport, regional land use, environmental conditions, and local disturbance patterns^60^. This study identified wind dispersal (affecting 15 PAs), and animal and bird migration (9 PAs for each) and tourism (8 PAs) as the dominant pathways in Egyptian protected areas, highlighting the interplay of natural and anthropogenic vectors. Species possessing traits suited to these pathways (e.g., wind-adapted microsclerochores, epizoochorous seeds hitchhiking on tourists) demonstrate clear advantages. The limited recorded impact of trade (3 PAs) and transportation (2 PAs) may stem from methodological challenges in tracking indirect pathways or localized biosecurity effectiveness.

Crucially, no statistically significant linear correlation was found between the number of pathways per PA and alien species richness (r = 0.272, p = 0.326). While a weak positive trend existed, this non-significance indicates that simply counting pathway types is less predictive than other factors: Pathway Intensity/Propagule Pressure: The volume or frequency of introductions via a single pathway (e.g., high tourist traffic) likely outweighs the presence of multiple low-intensity pathways^59^. Pathway Effectiveness: Pathways delivering large numbers of viable propagules, or propagules pre-adapted to local conditions, have greater impact. Environmental and Biotic Filters: Post-introduction establishment is governed by abiotic (climate suitability, disturbance, soil) and biotic (native competition, mutualists/pathogens) filters^61^. Other Key Drivers: Alien richness is likely driven more strongly by: Habitat Disturbance: Creating opportunities for establishment. Total Propagule Pressure: Combined number and frequency of introductions. Residence Time: Duration of introduction pressure. Connectivity: Proximity to source populations (e.g., urban/agricultural areas). PA Characteristics: Size, management effectiveness, surrounding land use, native diversity.

**Plate 1:**
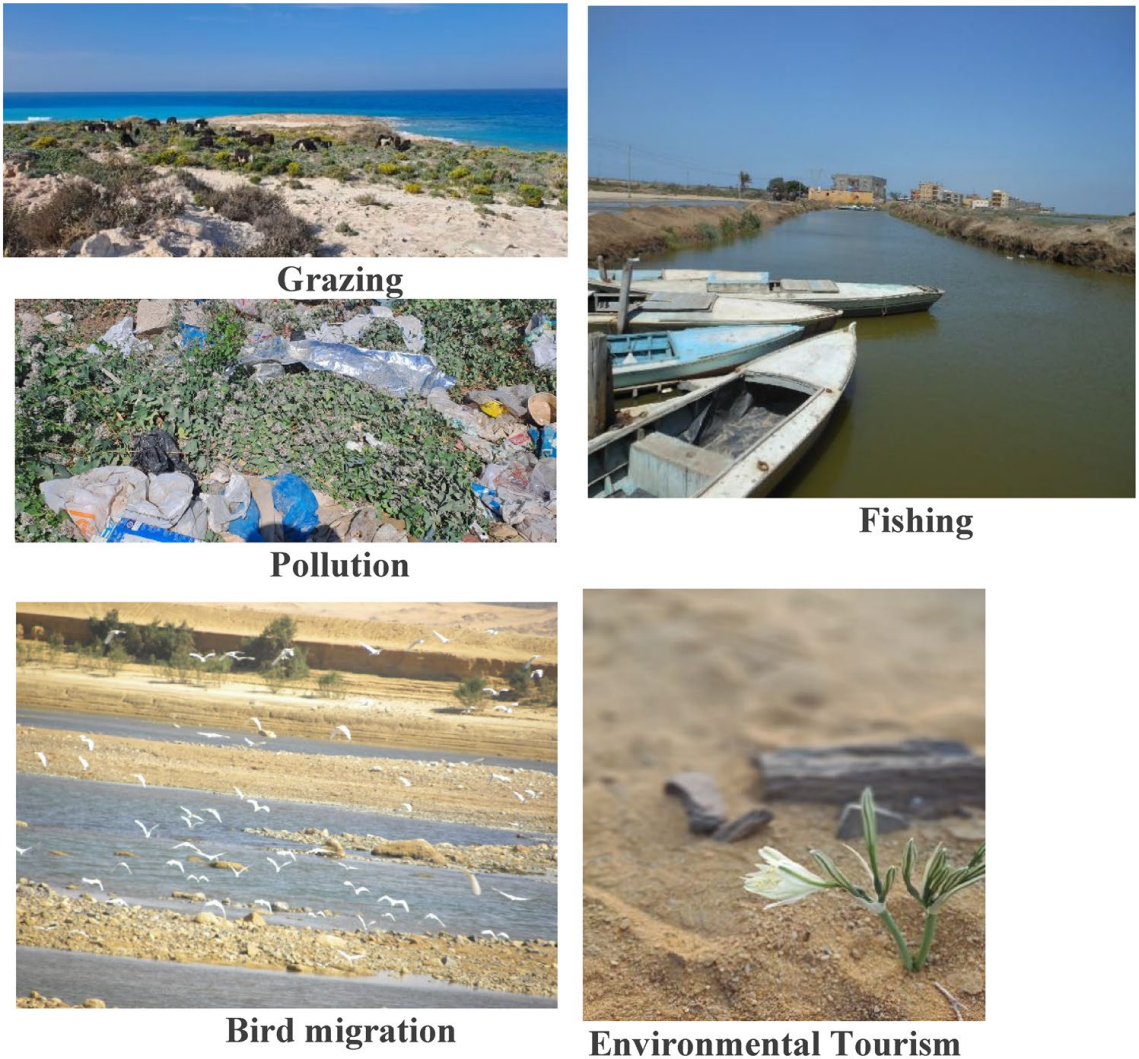
Pathways for the introduction of alien species in Egypt.

This study primarily identified pathways via field observations, assessing PA proximity to human influences and threats. Future research should track the specific entry pathway for individual species to enhance accuracy. The dominance of tourism-mediated dispersal underscores the need for targeted visitor education and strict entry-point inspections. Geographic disparities in alien richness (e.g., hotspots like Saint Katherine) necessitate tailored management plans addressing region-specific threats like overgrazing or water diversion (Plate. 1).

Management of alien plant invasions in Egyptian PAs is generally inadequate. The extent to which management interventions can be implemented is limited due to both the scarcity of resources for PAs, and competition for allocation of resources within overall park management programmes^62^. For example, resources may need to be allocated to anti-poaching, fire management, animal capture and relocation, monitoring of threatened species, infrastructure maintenance, community relations and invasive species management. Additionally, the lack of capacity, and awareness of the problem, and PA risk assessments to priorities threats undermines the importance of invasive species management^63^. Where control operations are in place, objective management effectiveness evaluations would assist in continuous improvement. Innovative approaches are required if progress is to be made in the long term. It is also highly unlikely that one management approach will suit all situations. However, examples do exist that may be tailored to provide unique solutions^50^**.**

## Conclusion

In conclusion, this study reveals a concerning level of alien species diversity within Egyptian protected areas, with significant implications for conservation management. Targeted monitoring and control efforts are crucial, particularly in areas with high alien species richness and a high proportion of naturalized and invasive species.

The findings of this research underscore the urgent need for proactive conservation strategies within EPAs. The high proportion of alien species, coupled with the dominance of invasive-prone families like Poaceae and Amaranthaceae, suggests a significant threat to native biodiversity and ecosystem function. Effective management interventions, including stricter regulations on tourism activities and enhanced monitoring, are essential to curb the introduction and spread of these species. Further research should prioritize assessing the long-term ecological impacts of these invasions and developing targeted control measures to preserve the unique biodiversity of these protected areas. Moreover, future research direction should focus on assessing the ecological impacts of these alien species and developing effective strategies to mitigate their spread and minimize their negative consequences on native biodiversity. Finally, our results confirm that the effectiveness of protected areas in preserving native biodiversity enhances resistance to invasion. However, their high biological value also makes them potential targets for invasive species, in addition to the fact that some improper human practices may contribute to increased invasion rates. This highlights the need for proactive management and continuous monitoring of these species within protected areas to maintain their ecological integrity and this is precisely the aspect we emphasize.

## Supplementary Information

Supplementary Information 1.

Supplementary Information 2.

## Data Availability

Available as supplementary.

## References

[1] Barbier, E., Knowler, D., Gwatipedza, J., Reichard, S. & Hodges, A. Implementing policies to control invasive plant species. *Bioscience* **63**, 132–138. 10.1525/bio.2013.63.2.9 (2013).

[2] Barral, A. Plant ecology Invasive species like it hot. *Nat. Plants* **5**, 645–645. 10.1038/s41477-019-0483-z (2019). 31263245 10.1038/s41477-019-0483-z

[3] Molano-Flores, B. An invasive plant species decreases native plant reproductive success. *Nat. Areas J.* **34**, 465–469. 10.3375/043.034.0408 (2014).

[4] Schaal, B., Gaskin, J. & Caicedo, A. Phylogeography, haplotype trees, and invasive plant species. *J. Hered.* **94**, 197–204. 10.1093/jhered/esg060 (2003). 12816959 10.1093/jhered/esg060

[5] Alidoost Salimi, P. et al. A review of the diversity and impact of invasive non-native species in tropical marine ecosystems. *Marine Biodivers. Rec.* **14**, 11. 10.1186/s41200-021-00206-8 (2021).

[6] Shaltout SK (2020). Assessment and documentation of the alien species in the Egyptian flora. Ph.D. Thesis, Faculty of Science Tanta University Tanta 263.

[7] Dudley N (ed) (2008) Guidelines for applying protected area management categories. IUCN, Gland. http://data.iucn.org/dbtw-wpd/edocs/PAPS-016.pdf

[8] Chape, S. et al. Measuring the extent and effectiveness of protected areas as an indicator for meeting global biodiversity targets. *Phil. Trans. Biol. Sci.* **360**, 443–455 (2005). 10.1098/rstb.2004.1592 PMC1569446 15814356

[9] Gaston, K. J., Jackson, S. F. & Cantú-Salazar, L. The ecological performance of protected areas. *Annu. Rev. Ecol. Evol. Syst.* **39**(1), 93–113 (2008).

[10] UNEP-WCMC and IUCN Protected planet: The World Database on Protected Areas (WDPA) and World Database on Other Effective Area-based Conservation Measures (WD-OECM) [Online] April 2025 Cambridge UK: UNEP-WCMC and IUCN. Available at: www.protectedplanet.net 2025

[11] Erwin, T. L. An evolutionary basis for conservation strategies. *Science* **253**, 750–752 (1991). 17835489 10.1126/science.253.5021.750

[12] El-Khalafy, M. M., Ahmed, D. A., Shaltout, K. H., Al-Sodany, Y. M. & Haroun, S. A. Re-assessment of the endemic taxa in the Egyptian Flora. *Afr. J. Ecol.* **59**, 784–796. 10.1111/aje.12880 (2021).

[13] Amer WM (2021) The worst invasive species to Egypt. invasive alien species.

[14] Galal, T. M., Dakhil, M. A., Hassan, L. M. & Eid, E. M. Population dynamics of Pistia stratiotes L. *Rendiconti Lincei-Scienze Fisiche E Naturali* **30**, 367–378. 10.1007/s12210-019-00800-0 (2019).

[15] Naguib, N. M., Awad, A. A., Barakat, O. E. & Higazy, A. M. Cyanodiveristy in some Egyptian protected areas. *Int. J. Adv. Res.* **2**, 1079–1096 (2014).

[16] Holmgren, P. K., Holmgren, N. H. & Barnett, L. C. *Index Herbar-iorum Part I: The Herbaria of The World, Regnum vegetabile 120* 8th edn. (New York Botanical Garden, 1990).

[17] Abd El-Hameed, R. M., Elias, W. A. & Abdel Rady, H. A. W. Assessing of sustainability practices in protected areas in Egypt in light of IUCN green list standard. *Minia J. Tour. Hospitality Res. MJTHR* **17**(2), 101–122 (2024).

[18] Arpa, N. Y. & Ceran, Y. Ecotourism, protected areas and nature conservation. *Fresenius Environ. Bull.* **24**, 250–257 (2015).

[19] Kurczewski, R. Ecotourism in protected areas - Chances and threats. *Ekol. Bratisl.* **20**, 403–407 (2001).

[20] Park, G. N. the NZ protected natural area program - scientific resources for a national cultural goal. *N. Z. J. Ecol.* **8**, 151–151 (1985).

[21] Picone, F., Buonocore, E., Chemello, R., Russo, G. F. & Franzese, P. P. Exploring the development of scientific research on marine protected areas: from conservation to global ocean sustainability. *Ecol. Inf.*10.1016/j.ecoinf.2020.101200 (2021).

[22] Rodriguez-Lopez, N., Dieguez-Castrillon, M. I. & Gueimonde-Canto, A. Sustainability and tourism competitiveness in protected areas: state of art and future lines of research. *Sustainability*10.3390/su11226296 (2019).

[23] Naughton-Treves, L., Holland, M. B. & Brandon, K. The role of protected areas in conserving biodiversity and sustaining local livelihoods. *Annu. Rev. Environ. Resour.* **30**, 219–252. 10.1146/annurev.energy.30.050504.164507 (2005).

[24] Yousef, W. M. & Elmansoury, A. A. sustainable architectural design in the egyptian natural protected areas - al-gharqana fishermen village, nabq protected area, sharm el sheikh Egypt. *J. Eng. Sci. Technol.* **16**, 2980–3004 (2021).

[25] Harhash, K. A. et al. Conservation oriented habitat classification scheming and mapping of Egypt. *Environ. Syst. Res.* **4**, 8. 10.1186/s40068-015-0034-1 (2015).

[26] EEAA Protected areas of Egypt: Towards the future. nature conservation sector, egyptian environmental affairs agency, ministry of state for environmental affairs, Cairo Egypt 2006

[27] El-Beheiry, M., Hosni, H., Sharaf El-din, A., Shaltout, S. & Ahmed, D. Updating the checklist of the alien flora in Egypt. *Taeckholmia* **40**(1), 41–56. 10.21608/taec.2020.21300.1011 (2020).

[28] Täckholm V, Drar M (1950-1969) Flora of Egypt: Vols. II, III and VI, Bull. Fac. Sci. no. 28 30, 36:537 644, 423.

[29] Täckholm V Students’ flora of Egypt: 2nd ed Cairo University. 888 (1974)

[30] El–Hadidi, M. N. and Fayed, A. Material for excursion flora of Egypt (EFE). Taeckholmia 15: 1-233 (1994/ 95)

[31] El–Hadidi, M. N. (ed.) Flora Aegyptiaca: Volume one Parts 1&2. Palm Press Cairo. 170 (2000)

[32] Boulos, L. Flora of Egypt: volume 1-5 (Azollaceae-Oxalidaceae). Al–Hadara Publishing, Cairo. (1999-2005)

[33] Boulos, L. *Flora of Egypt Checklist Revised* Annotated. (Al-Hadara Publishing, 2009).

[34] Dobignard & Chatelain Synonymic index of the flora of North Africa Volume 1-5 (2010-2013)

[35] Hassan, L. M. Plant life in the digla conserved area, hyperarid desert Egypt. *J. Biol. Sci.* **2**, 533–537 (2002).

[36] Shaltout, K., Ali, M., Hassan, L. & Galal, T. Habitat and vegetation of Lake Edku Egypt. *Taeckholmia* **25**(1), 61–90. 10.21608/taec.2005.12304 (2005).

[37] Khalil, M. T. and Shaltout, K. H. (2006). Lake Bardawil; Zaranik Protected Area. Publication of National Biodiversity Unit No. 15 Egyptian Environmental Affairs Agency Egypt 580 (2006)

[38] Amin S. A. Ecological study on the plant life in WadiEl-Rayan area El-Fayoum Egypt. PhD thesis, Ecology Cairo Univ Fac. of Sci (1998)

[39] Amin S. A. Phytosociological study of Lake QARUN, El-Fayoum, Egypt. Msc thesis Ecology Cairo Univ, Fac. of Sci (1992)

[40] Baayo K. A. Phytosociological and floristic studies on Sallum area Egypt Assiut University. (2005)

[41] El-Saied, A., El-Ghamry, A., Khafagi, O.-M.A., Powell, O. & Bedair, R. Floristic diversity and vegetation analysis of Siwa Oasis: An ancient agro-ecosystem in Egypt’s Western Desert. *Sci. Rep.* **60**, 361–372 (2015).

[42] Abdel-Ghaffar, A. S., Marei, S. M. & Gaber, H. M. (eds.) *Integrated land development of Southern Egypt: available resources and alternative options*. (Alexandria University, **1998**)

[43] Abutaha, M. M., El-Khouly, A. A., Jürgens, N. & Oldeland, J. Plant communities and their environmental drivers on an arid mountain, Gebel Elba Egypt. *Veg. Classifi. Surv.* **1**, e38644. 10.3897/VCS/2020/38644 (2020).

[44] Alhobishi, H. A. Flora and vegetation of wadi degla protected area, North Eastern Desert, Egypt. Ph.D. thesis, Faculty of Science, Menoufia University, Menoufia, Egypt. (2024)

[45] Csiszár, Á., Kézdy, P., Korda, M. & Bartha, D. Occurrence and management of invasive alien species in Hungarian protected areas compared to Europe. *Folia Oecologica* **47**(2), 178–191. 10.2478/foecol-2020-0021 (2020).

[46] Richardson, D. et al. Naturalization and invasion of alien plants: concepts and definitions. *Divers. Distrib.* **6**, 93–107 (2000).

[47] Dansereau, P. & Lems, K. The grading of dispersal types in plant communities and their ecological significance. *Institut Botanique de L’Universitè de Montrèal, Montrèal* **71**, 1–52 (1957).

[48] Harrower, C. A., Scalera, R., Pagad, S., Schönrogge, K., & Roy, H. E. *Guidance for interpretation of the categories on introduction pathways under the convention on biological diversity*. Subsidiary Body on Scientific, Technical and Technological Advice (SBSTTA), Twenty-second meeting, Montreal, Canada, 2–7 July 2018. CBD/SBSTTA/22/INF/1. Convention on Biological Diversity. (2018)

[49] Pashkevych, N. & Burda, R. Distribution of alien species from Poaceae and Asteraceae families in the protected areas of Ukrainian forest-steppe – Thaiszia –. *J. Bot.* **27**(1), 029–039 (2017).

[50] Foxcroft, L. C. et al. Chapter 2: The bottom line: impacts of alien plant invasions in protected areas. In *Plant invasions in protected areas: patterns, problems and challenges* (eds Foxcroft, L. C. et al.) 19–41 (Springer, 2014).

[51] Braun, M., Schindler, S. & Essl, F. Distribution and management of invasive alien plant species in protected areas in Central Europe. *J. Nat. Conserv.* **33**, 48–57 (2016).

[52] El-Barougy, R. F. et al. Invasion risk assessment using trait-environment and species distribution modelling techniques in an arid protected area: Towards conservation prioritization. *Ecol. Indic. Elsevier.* **129**(2021), 107951 (2021).

[53] Dudley N, Stolton S, Belokurov A et al Natural solutions: protected areas helping people cope with climate change. WWF International, Gland (2010)

[54] Simberloff, D. et al. Impacts of biological invasions: what’s what and the way forward. *Trends. Ecol. Evol.* **28**, 58–66 (2013). 22889499 10.1016/j.tree.2012.07.013

[55] Spear, D. et al. Human population density explains alien species richness in protected areas. *Biol. Conserv.* **159**, 137–147 (2013).

[56] Blackburn, T. M. et al. A proposed unified framework for biological invasions. *Trends Ecol. Evol.* **26**(7), 333–339 (2011). 21601306 10.1016/j.tree.2011.03.023

[57] Hulme, P. E. Trade, transport and trouble: managing invasive species pathways in an era of globalization. *J. Appl. Ecol.* **46**(1), 10–18 (2009).

[58] Butchart, S. H. M. Global biodiversity: indicators of recent declines. *Science* **328**, 1164–1168. 10.1126/science.1187512 (2010). 20430971 10.1126/science.1187512

[59] Essl, F. et al. Historical legacies accumulate to shape future biodiversity in an era of rapid global change. *Divers. Distrib.* **21**, 534–547 (2015).

[60] Seipel, T. et al. Processes at multiple spatial scales determine non-native plant species richness and similarity in mountain regions around the world. *Glob. Ecol. Biogeogr.* **21**, 236–246 (2012).

[61] Raudsepp-Hearne, C. et al. Untangling the environmentalist’s paradox: why is human well-being increasing as ecosystem services degrade?. *BioScience* **60**, 576–589 (2010).

[62] De Poorter M Invasive alien species and protected areas: a scoping report. Part 1. Scoping the scale and nature of invasive alien species threats to protected areas, impediments to invasive alien species management and means to address those impediments. Global Invasive Species Programme, Invasive Species Specialist Group. http://www.issg.org/gisp_publications_reports.htm (2007)

[63] Genovesi P, Scalera R, Brunel S et al Towards an early warning and information system for invasive alien species (IAS) threatening biodiversity in Europe. EEA technical report n.5/2010. European Environment Agency Copenhagen (2010)

