# Supplementary material for: Assessment taxonomy and pathways of alien plant species in Egyptian protected areas
Source: Sci Rep. 2025 Nov 12;15:39577. doi: 10.1038/s41598-025-24266-3 (PMC12612076; doi:10.1038/s41598-025-24266-3)
Supplement: Supplementary file 1 — Supplementary Information 1. [file 41598_2025_24266_MOESM1_ESM.docx]

Appendix. Questionnaire about invasive alien species and protected areas in Egypt. The same content via (Csiszár et al 2020)

1. General information First name:

Family name:

Email address:

Country:

Name of protected area:

WDPA ID:

The WDPA ID is the unique identification number assigned by UNEPWCMC to each marine or terrestrial protected area. You can find the WDPA ID of your protected area at <http://www.wdpa.org/>

<http://www.wdpa.org/>

1. What in your opinion are the most important threats to your protected area? (Rank 1 is the most important; please tick at

least 3 responses).

|  | rank 1 | rank 2 | rank 3 | rank 4 | rank 5 |
| --- | --- | --- | --- | --- | --- |
| Pollution | □ | □ | □ | □ | □ |
| Overgrazing | □ | □ | □ | □ | □ |
| Erosion | □ | □ | □ | □ | □ |
| Overexploitation | □ | □ | □ | □ | □ |
| Habitat loss/fragmentation | □ | □ | □ | □ | □ |
| Poaching | □ | □ | □ | □ | □ |
| Tourism | □ | □ | □ | □ | □ |
| Invasive species | □ | □ | □ | □ | □ |
| Waste | □ | □ | □ | □ | □ |
| Other  Specify other | □ | □ | □ | □ | □ |

1. Is there a checklist of alien animals present in your protected area?

□ Yes □ No

Partial (please specify what kind of list).

It is very important to specify if you have only partial checklists (e.g. only invasive animals, only some taxonomic groups, etc.).

1. Top invasive species (most harmful) in your protected area: ANIMALS. (Please indicate at least 1 species). Species 1

Species 2

Species 3

Species 4

Species 5

1. What are the kinds of management activities of the top invasive ANIMALS being implemented in your protected area? (Please tick at least 1 response).

Monitoring & surveillance

Prevention Control Eradication

Habitat restoration

Native species recovery

Communication Education

Public involvement

None

Species 1 □ □ □ □ □ □ □ □ □ □

Species 2 □ □ □ □ □ □ □ □ □ □

Species 3 □ □ □ □ □ □ □ □ □ □

Species 4 □ □ □ □ □ □ □ □ □ □

Species 5 □ □ □ □ □ □ □ □ □ □

1. Is there a checklist of alien plants present in your protected area?

□ Yes □ No

Partial (please specify what kind of list)

It is very important to specify if you have only partial checklists (e.g. only invasive plants, only some taxonomic groups, etc.).

1. Top invasive species (most harmful) in your protected area: PLANTS. (Please indicate at least 1 species). Species 1

Species 2

Species 3

Species 4

Species 5

1. What are the kinds of management activities of the top invasive PLANTS being implemented in your protected area? (Please tick at least 1 response).

Monitoring & surveillance

Prevention Control Eradication

Habitat restoration

Native species recovery

Communication Education

Public involvement

None

Species 1 □ □ □ □ □ □ □ □ □ □

Species 2 □ □ □ □ □ □ □ □ □ □

Species 3 □ □ □ □ □ □ □ □ □ □

Species 4 □ □ □ □ □ □ □ □ □ □

Species 5 □ □ □ □ □ □ □ □ □ □

1. What in your opinion are the worst impacts caused by invasive species in your protected area? (Rank 1 is the worst; please

tick at least 1 response).

|  | rank 1 | rank 2 | rank 3 | rank 4 | rank 5 |
| --- | --- | --- | --- | --- | --- |
| Competing with native species | □ | □ | □ | □ | □ |
| Predating native species | □ | □ | □ | □ | □ |
| Transmitting diseases to native species | □ | □ | □ | □ | □ |
| Hybridising with native species | □ | □ | □ | □ | □ |
| Affecting habitats | □ | □ | □ | □ | □ |
| Ecosystem changes (trophic level, fire regime, hydrology, etc.) | □ | □ | □ | □ | □ |
| Affecting human health (vector of diseases, allergenic, toxic, etc.) | □ | □ | □ | □ | □ |
| Damaging infrastructures | □ | □ | □ | □ | □ |
| Damaging landscape | □ | □ | □ | □ | □ |
| Damaging agriculture/forestry/aquaculture | □ | □ | □ | □ | □ |
| Other | □ | □ | □ | □ | □ |
| Specify other |  |  |  |  |  |

1. What in your opinion are the most effective management options to deal with the spread of invasive species in your protected area? (Rank 1 is the most effective; please tick at least 1 response).

|  | rank 1 | rank 2 | rank 3 | rank 4 | rank 5 |
| --- | --- | --- | --- | --- | --- |
| Prevention | □ | □ | □ | □ | □ |
| Control | □ | □ | □ | □ | □ |
| Eradication | □ | □ | □ | □ | □ |
| Habitat restoration | □ | □ | □ | □ | □ |
| Native species recovery | □ | □ | □ | □ | □ |
| Communication | □ | □ | □ | □ | □ |
| Education | □ | □ | □ | □ | □ |
| Regulatory | □ | □ | □ | □ | □ |
| Public involvement | □ | □ | □ | □ | □ |
| Other  Specify other | □ | □ | □ | □ | □ |

1. What in your opinion are the key impediments in dealing with the spread of invasive species in your protected area? (Rank 1 is the most important; please tick at least 1 response).

|  | rank 1 | rank 2 | rank 3 | rank 4 | rank 5 |
| --- | --- | --- | --- | --- | --- |
| Limited resources (financial/staff) | □ | □ | □ | □ | □ |
| Lack of capacity | □ | □ | □ | □ | □ |
| Lack of awareness | □ | □ | □ | □ | □ |
| Lack of information | □ | □ | □ | □ | □ |
| Public/stakeholders opposition to management | □ | □ | □ | □ | □ |
| Legal impediments | □ | □ | □ | □ | □ |
| Institutional impediments | □ | □ | □ | □ | □ |
| Other  Specify other | □ | □ | □ | □ | □ |

1. Do you have any comments about the survey?

Thank you very much for participating in the survey!
